# Body composition in young female eating-disorder patients with severe weight loss and controls: evidence from the four-component model and evaluation of DXA

**DOI:** 10.1038/ejcn.2015.111

**Published:** 2015-07-15

**Authors:** J C K Wells, D Haroun, J E Williams, D Nicholls, T Darch, S Eaton, M S Fewtrell

**Affiliations:** 1 Childhood Nutrition Research Centre, UCL Institute of Child Health, London, UK; 2 Department of Psychological Medicine, Child and Adolescent Mental Health, Great Ormond Street Hospital, London, UK; 3 Developmental Biology and Cancer Programme, UCL Institute of Child Health, London, UK

## Abstract

**Background/Objectives:**

Whether fat-free mass (FFM) and its components are depleted in eating-disorder (ED) patients is uncertain. Dual energy X-ray absorptiometry (DXA) is widely used to assess body composition in pediatric ED patients; however, its accuracy in underweight populations remains unknown. We aimed (1) to assess body composition of young females with ED involving substantial weight loss, relative to healthy controls using the four-component (4C) model, and (2) to explore the validity of DXA body composition assessment in ED patients.

**Subjects/Methods:**

Body composition of 13 females with ED and 117 controls, aged 10–18 years, was investigated using the 4C model. Accuracy of DXA for estimation of FFM and fat mass (FM) was tested using the approach of Bland and Altman.

**Results:**

Adjusting for age, height and pubertal stage, ED patients had significantly lower whole-body FM, FFM, protein mass (PM) and mineral mass (MM) compared with controls. Trunk and limb FM and limb lean soft tissue were significantly lower in ED patients. However, no significant difference in the hydration of FFM was detected. Compared with the 4C model, DXA overestimated FM by 5±36% and underestimated FFM by 1±9% in ED patients.

**Conclusion:**

Our study confirms that ED patients are depleted not only in FM but also in FFM, PM and MM. DXA has limitations for estimating body composition in individual young female ED patients.

## Introduction

Eating disorders (ED) involving severe weight loss have a lifetime prevalence in women of 2%–4% in Western industrialized populations, with adolescents most at risk.^1^ Patients with anorexia nervosa (AN) and some atypical ED are characterized by loss of fat mass (FM), which has important physiological consequences. However, the literature on fat-free mass (FFM) depletion and its components is inconsistent, in particular the extent to which muscle wasting occurs in these patients.

Some studies found lower FFM in AN patients compared with controls,^2, 3, 4, 5, 6^ whereas others reported no difference.^7, 8, 9, 10, 11^ This inconsistency also applies to other diseases of malnutrition. In dialysis patients with chronic renal failure, some studies found reduced FFM compared with controls,^12, 13^ whereas another study found no difference.^14^ Cystic fibrosis patients had lower FFM than controls in adolescence, but not in childhood.^15, 16^

Some studies have further reported that FFM components, including mineral^2, 17, 18, 19^ and protein mass (PM),^2, 4, 20, 21^ are significantly reduced in AN patients. PM declines in proportion with disease severity;^4, 21^ however, the protein component of FFM may be rapidly replenished after weight regain.^20^ In other diseases of malnutrition, PM was also found to be depleted; however, this was not reflected by simple anthropometric measurements.^22, 23, 24^

Body mass index (BMI) is the most widely used measure of nutritional status in ED patients and gains in BMI during treatment are considered a sign of improved health. However, BMI is a poor index of fatness in females with ED, explaining only ~50% of the variance in percentage fat estimated by skinfolds or densitometry.^25^ Change in BMI was also reported to be a poor indicator of body composition change in such patients.^26^ Other techniques such as dual-energy X-ray absorptiometry (DXA) are increasingly used; however, accuracy of DXA in healthy children and adolescents is imperfect^27, 28^ and its validity in ED patients has been evaluated only against skinfold thickness and BMI,^29^ which are not suitable reference methods.

The primary aim of this study was to compare body composition between adolescent girls with EDs and healthy controls. We used the four-component (4C) model, the ‘gold standard' for assessing tissue masses, as it minimizes theoretical assumptions concerning the properties of FFM and hence measures FM more accurately. It also allows possible differences in PM and mineral mass (MM) to be examined. The secondary aim was to evaluate the utility of BMI and DXA for estimating body composition in ED patients, using the 4C model as the reference method.

## Subjects and methods

### Subjects

Subjects included 13 female ED patients involving substantial weight loss and 117 female controls, aged between 10 and 18 years. The patients were recruited from a specialist clinic at Great Ormond Street Hospital, London, a tertiary referral hospital. All had been diagnosed previously and were referred either because of clinical deterioration or because, despite medical stabilization, they were not responding to treatment. All patients met full criteria for AN at some point and would therefore be diagnosed with AN using DSM5 criteria.

Controls were recruited from an ongoing body composition research project for healthy children and adolescents. Controls were healthy, born full-term (>37 weeks gestation) and had never taken any medication affecting their growth or nutritional status. They also had no history of EDs. The study was approved by the Research Ethics Committee of UCL Institute of Child Health/Great Ormond Street Hospital. All subjects over 16 years of age gave written consent before participating in the study; parents or guardians gave written consent for younger subjects, who also gave verbal assent.

### Anthropometry

Body weight was measured with minimum clothing to 0.01 kg using an electronic scale integral to the air-displacement plethysmography instrumentation (see below). Height was measured to 1 mm using a wall-mounted stadiometer (Holtain, Dyfed, UK). BMI was calculated as weight/height^2^. Circumferences of the mid-upper arm, waist, hips, thigh and calf were also measured using a non-stretchable flexible tape. Height, weight, BMI and waist circumference were converted into s.d. scores using current UK 1990 reference data.^30, 31^ Pubertal status was self-assessed using line drawings.

### Deuterium oxide dilution

Total body water (TBW) was determined by deuterium oxide, using an oral dose equivalent to 0.05 g of 99.9 atom% deuterium-labelled water per kg body weight. Saliva samples were collected using cotton wool swabs at baseline and 4 h after dosing. Isotopic enrichment of saliva samples was determined by isotope-ratio mass spectrometry.^32^ Raw TBW was corrected for any drinks consumed during the equilibration period.

### Air-displacement plethysmography

Body volume was measured by air-displacement plethysmography using BODPOD instrumentation (Life Measurement Instruments, Concord, CA, USA). The subjects wore a close-fitting swimming costume and swimming cap. Two complete tests (that is, a minimum of four raw volume measurements) were performed on each subject.^32^ Lung volume was predicted using age- and sex-specific equations.^33^

### Dual-energy X-ray absorptiometry

Bone mineral content was measured using DXA (Lunar Progidy, Madison, WI, USA). The subjects removed objects and clothes containing metal before undergoing the scan. Subjects lay supine, with arms and legs at their sides. The typical scan duration was 10–15 min, depending on height. The estimated radiation exposure was 2.2 μSv per scan. Data on whole body and regional FM, bone mineral content and non-bone lean soft tissue were extracted.

### Four-component model

The 4C model differentiates weight into FM, water, protein and mineral. Using the raw measurements described above, FM can be calculated using the following equation:^34^

FM (kg)=2.747 (body volume)−0.71 (TBW)+1.46 (bone mineral content)−2.05 (weight)

FFM was calculated as: weight−FM

To aid initial comparison of patients and controls, FM and FFM were adjusted for height, by calculating the FM index (FMI=FM/height^2^) and FFM index (FFMI=FFM/height^2^). Hydration of FM (*H*_FFM_) was calculated as TBW/FFM. Total body MM was calculated as bone mineral content × 1.2741.^34^ PM was calculated as FFM−(MM+TBW). The protein:mineral ratio indicates the degree of mineralization of fat-free tissue.

A Hattori plot^35^ was generated to express the relative contributions of FFMI versus FMI to BMI in the ED patients and controls. Whole-body FM and FFM by the 4C model, and regional fat and lean soft tissue by DXA, were expressed as *z*-scores relative to the UK reference data.^33^

### Sample size and statistical analysis

We aimed to recruit 16 ED patients, which would be adequate to identify a 1 s.d. difference in any outcome relative to 16 controls. However, to increase statistical power of the comparison and to be able to address a wide range of size and pubertal status in the controls, we analyzed all available data from a study of healthy girls, which have been published as the UK body composition reference data.^33^

All statistical analyses were performed using Statistical Package for the Social Sciences (SPSS v.15 for Windows, SPSS Inc, Chicago, IL, USA). Independent sample *t*-tests and *χ*^2^-tests were used to test for differences in background characteristics, age and anthropometry between the patients and controls.

General linear models were used to test for differences in body circumferences, FM, FFM and the various components of FFM including water, protein and mineral, and regional body composition (by DXA) between patients and controls, adjusting for potential confounding factors (age, height and pubertal stage). To express differences in percentage terms, raw data were log-transformed and the regression coefficients multiplied by 100%.

Pearson's correlations were used to investigate associations between BMI and body composition estimated by the 4C model (FMI and FFMI) in the patients and controls. Bland–Altman analyses were used to assess the bias in body composition measurements of the ED patients by DXA, using 4C data as the reference method.^36^ Limits of agreement between measurements were expressed as ±2 s.d. of the mean bias. The correlation between the bias and mean of measured values was also determined. The bias was expressed as percent of the mean by natural log transformation of the bias of individual values and multiplying by 100.

## Results

The control girls and ED patients were all White Europeans, except for one ED patient who was Indian. This patient did not significantly differ in age or any of the anthropometric or body composition measurements compared with the rest of the patients; therefore, her data were included in the analyses. All subjects had data for the 4C model, except for one ED patient who only had a DXA scan. Therefore, the main comparisons of ED versus controls, and of the accuracy of DXA, involved 12 ED patients and 117 controls; however, an additional ED data point was available for the regional DXA comparison.

Characteristics of the ED patients and controls are presented in Table 1. The patients were aged between 10 and 18 years with a mean of 15.3±1.9 years. Control girls were also aged between 10 and 18 years, but were on average 1.6 years younger than those with EDs (*P*=0.006) and had earlier pubertal stage (3.2, s.d 1.3 vs 3.6, s.d 1.4), significant by *χ*^2^-test (*P*=0.007). As expected, the ED group had significantly lower weight and BMI s.d. scores (*P*<0.001) than the controls. The controls were 2.9 cm taller, equivalent to 0.4 s.d. scores, but not significantly so (*P*=0.2). The mean BMI s.d. scores of 0.34 (s.d. 1.19) in the controls indicate that they are representative of British girls in general.^33^

In terms of background characteristics, there were no significant differences for birth weight and parental BMI between patients and controls. However, all anthropometric measurements differed significantly between the groups, including body circumferences.

**Figure 1:**
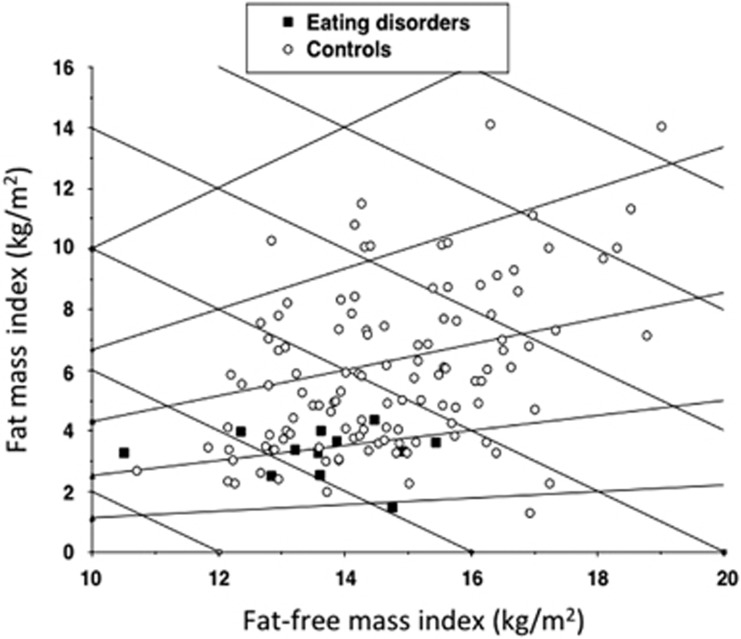
A Hattori plot illustrating differences in FMI and FFMI by the 4C model between ED patients and controls. As FMI and FFMI add up to BMI, downward-crossing diagonal lines express constant BMI values. The ED patients have reduced FMI and FFMI relative to the control population.

Table 2 presents the body composition outcomes. The only statistically significant differences were lower body volume, FM, FFMI and FMI in the ED patients. The s.d. scores for FM and FFM were also significantly lower in the patients. Figure 1 shows a Hattori plot of FMI versus FFMI in ED patients and controls. The ED patients were all low on the graph, confirming their uniformly low FMI compared with the controls. They were also grouped towards the left-hand side of the graph, indicating a lower range of FFMI. However, due to the difference between groups in mean age, further statistical adjustments were required to test for differences in regional body composition and FFM composition. The hydration value of 72.8% gave no indication of edema in the ED group.

After adjusting for height and age, the ED patients had mean deficits of 20.4% in mid-upper arm girth, 11.1% in waist girth, 11.7% in hip girth, 14.9% in thigh girth and 11.0% in calf girth. These data indicate a greater depletion of upper arm and upper leg tissue compared with abdominal regions.

Table 3 illustrates crude whole-body composition differences between ED patients, after adjusting for differences in age and finally after adjusting for differences in age, pubertal status and height. In all models, ED patients had significantly lower FM, FFM, MM and PM. After adjusting for age, height and puberty, the deficits compared with controls were equivalent to 51.9% in FM (*P*<0.0001), 10.8% in FFM (*P*<0.0001), 12.2% in MM (*P*<0.001) and 9.1% in PM (*P*<0.03). However, differences in hydration or density of FFM did not reach significance and no difference in the protein:mineral ratio was found.

Regional body composition, assessed by DXA, was also compared between ED patients and controls (Table 3). After adjusting for age, sex and pubertal stage, the percentage deficit in FM was 76.1%, 58.9% and 58.6% for the arm, leg and trunk, respectively. Equivalent deficits for the bone were 11.4%, 10.9% and 18.3%, and for lean soft tissue were 13.9%, 9.3% and 4.3%. As with body girths, these data indicate greater loss of fat in the arm compared with the trunk, and greater loss of lean tissue in the limbs compared with the trunk. Using the new UK reference data, mean s.d. scores of the patients were −1.41 (0.84) for arm fat, −1.57 (0.79) for arm lean, −1.60 (0.77) for leg fat, −1.35 (0.93) for leg lean, −1.14 (0.69) for trunk fat and −0.97 (0.93) for trunk lean, all *P*<0.02 (see Supplementary Table S1 for full details).

The utility of BMI and DXA for body composition assessment was then assessed using the 4C model as the reference method. In ED patients, the correlation between BMI and FMI estimated by the 4C model was low and not statistically significant (*r*=0.47; *P*=0.1), whereas in controls this correlation was highly significant (*r*=0.92; *P*<0.001). The correlation between BMI and FFMI in both groups was highly significant (ED patients: *r*=0.85; *P*<0.001; controls: *r*=0.76; *P*<0.001).

**Figure 2:**
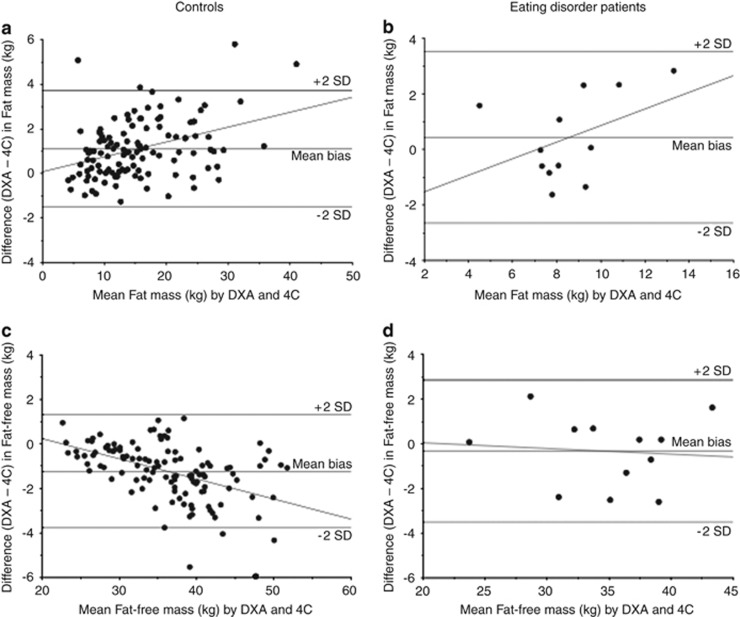
Bland–Altman plots to evaluate the accuracy of DXA for body composition assessment in ED patients. Bias in body composition (DXA value−4C value) on the *y* axis is plotted against body composition by both techniques. (**a**) Fat mass – controls; (**b**) Fat mass – ED patients; (**c**) Fat-free mass – controls; (**d**) Fat-free mass – ED patients.

The evaluation of the accuracy of DXA for body composition assessment is given in Table 4. In the ED patients, DXA was found to overestimate FM and underestimate FFM by 4.6% and 0.9%, respectively. Even though the bias was not very high, the limits of agreement for FM and FFM were ±36.4% and ±9.4%, respectively. Similarly, compared with the 4C model, DXA was found to overestimate FM and underestimate FFM in the control girls. The magnitude of the biases in FM and FFM did not differ significantly between ED patients and controls. A graphical illustration of the findings for the ED patients is shown in Figure 2.

## Discussion

Our study demonstrates that female adolescents with EDs involving substantial weight loss have reduced FM and FFM compared with controls, after adjusting for differences in age, height and pubertal stage. In addition, there were significant deficits in the protein and mineral components of FFM, although not of sufficient magnitude to indicate differences in the physical or biological properties of fat-free tissue. In addition to differences in whole-body composition, weight loss differed by region. ED patients had bone and FM deficits in their trunks and limbs, and lower lean soft tissue in their limbs. Although consistent with anthropometric data on girths, the magnitudes of these regional DXA soft tissue differences are less reliable than the equivalent whole-body 4C data, as we further demonstrated poor accuracy of DXA for estimating body composition, especially fatness, in ED patients.

Previous studies describing body composition differences between AN patients and controls have been inconsistent.^2, 3, 4, 5, 6, 7, 8, 9, 11^ This inconsistency may be due in part to the use of different body composition methods. DXA has been used in several studies of ED patients;^2, 5, 37^ however, these studies have not demonstrated the validity of DXA in ED patients using an accurate reference method. In healthy children and adolescents, the accuracy of DXA has been shown to vary across the range of nutritional status.^27, 28^

In the present study, the limits of agreement for DXA are similar in absolute terms in ED patients (±3 kg FM or FFM) to those in healthy subjects (±2.6 kg). In percentage terms, owing to the smaller tissue masses, these differences are relatively greater in ED patients (±36% FM and ±9% FFM). These findings support our earlier conclusion that DXA may have limitations for monitoring change in body composition in individuals gaining or losing tissue masses.^27^

PM is expected to correlate with height and, independently, to increase during pubertal maturation. An important component of our work was therefore to disentangle the greater age and more advanced pubertal stage of our patients relative to the controls, from their shorter height. After these adjustments, PM was significantly lower in the ED patients. This agrees with the findings from several previous studies of adolescents with EDs, which estimated total body nitrogen by *in-vivo* neutron activation analysis.^2, 20, 29^ *In-vivo* neutron activation analysis is used as an index of PM on the assumption that 98% of nitrogen is contained in protein.^38^ Accuracy of *in-vivo* neutron activation analysis for PM is estimated at 3%–7%^39^ however, a disadvantage of using *in-vivo* neutron activation analysis is that unlike the 4C model, it provides no data on other components of FFM. Protein depletion in children with chronic renal failure contributed to growth failure and severe stunting,^40^ which have also been described in patients with AN.^41, 42^ Therefore, the ability to monitor PM would be valuable in the clinical management of ED patients.

We also observed depleted MM in the ED patients, consistent with some previous studies,^43, 44^ although another study showed no significant deficit.^45^ Inconsistent findings between studies may be due to variability in the duration of illness, as the study with negative findings assessed bone mineral status in adolescents soon after the onset of AN, whereas we studied a heterogeneous sample of ED patients referred to a tertiary treatment centre, many of whom have had chronic or established illness despite their young age. More generally, the duration of malnutrition is considered an important predictor of bone loss.^46^

Most work on the nutritional status of ED patients has relied on weight and height indices. In our patients, only 22% of the variance in FMI could be predicted by BMI. This is consistent with findings from previous studies from healthy subjects^47^ and AN patients,^25^ showing that BMI does not accurately reflect body composition, especially fatness, and may be especially poor for monitoring disease progression and response to treatment.

The main limitation of our study is the small sample size for the ED patients and we did not manage to recruit our target of 16 patients. However, owing to the powerful effect of EDs on body composition and the large number of control subjects, we were able to detect statistically significant differences in almost all outcomes compared with healthy controls. Equally, our evaluation of DXA is complemented by previous work demonstrating differential bias across the entire range of nutritional status. Hence, despite the small sample size, our main conclusions are likely to be robust. Another limitation is the use of self-reported puberty status^48^ rather than physical examination. However, adjustment for puberty made little difference to the results, whereas size was more important.

In summary, our study using the gold standard 4C model demonstrates reduction in FM, FFM, PM and MM in adolescent ED patients, relative to healthy controls. Previously, FFM and PM have been shown to predict clinical outcome and morbidity during starvation and other illnesses of malnutrition. Hence, accurate measurements of body composition in ED patients may be important for monitoring the success of their treatment and in preventing future complications such as bone loss and infertility. A multi-component model may be more accurate for such longitudinal measurements than DXA.

## Figures and Tables

**Table 1:** Description of the sample: background characteristics, age and anthropometry

	*Eating disorders*			*Controls*		
	N	*Mean*	*s.d.*	N	*Mean*	s.d
Age (years)*	13	15.3	1.9	117	13.7	2.0
Birth weight (kg)	12	3.3	0.6	116	3.5	0.5
Maternal BMI (kg m^−2^)	12	25.7	5.3	112	25.3	5.4
Paternal BMI (kg m^−2^)	12	25.0	4.0	105	26.4	3.9
						
*Raw measures*						
Weight (kg)**	13	43.4	5.9	117	51.8	12.4
Height (m)	13	1.58	0.10	117	1.60	0.10
BMI (kg m^−2^)**	13	16.9	1.4	117	20.6	3.7
Weight SDS**	13	−1.4	1.0	117	0.4	1.1
Height SDS	13	−0.1	1.3	117	0.3	0.9
BMI SDS**	13	−1.5	1.0	117	0.3	1.2
						
*Circumferences (cm)*						
Mid-upper arm**	12	21.6	1.7	117	25.8	3.7
Hip*	12	82.1	5.8	117	88.6	11.0
Thigh**	12	44.7	3.4	116	50.7	6.9
Calf*	12	30.4	2.0	117	33.0	3.6
Waist**	12	62.9	2.7	117	69.1	8.0
Waist SDS**	11	−0.2	0.5	105	1.0	1.0

Abbreviations: BMI, body mass index; SDS, s.d. scores.

Groups compared by two-sample *t*-test. *P=0.05, **P<0.001.

**Table 2:** Body composition outcomes for the ED patients and controls

	*EDs*			*Controls*		
	N	*Mean*	s.d	N	*Mean*	s.d
*Raw measurements*						
Body volume (l)^a^	12	41.1	5.8	117	50.2	12.5
Total body water (l)	12	25.9	4.2	117	27.6	5.2
						
*4C model outcomes*						
FFM (kg)	12	35.4	5.6	117	36.9	7.1
FM (kg)^a^	12	7.9	1.9	117	14.8	7.1
FFM-SDS^a^	12	−1.13	1.02	117	0.02	1.02
FM-SDS^a^	12	−1.41	0.97	117	0.11	1.06
FFMI (kg m^−2^)^b^	12	13.8	1.3	117	14.7	1.6
FMI (kg m^−2^)^b^	12	3.1	0.8	117	5.9	2.6
*H*_FFM_ (%)	12	72.8	0.9	117	74.6	1.6
*D*_FFM_ (kg/l)	12	1.097	0.004	117	1.096	0.006
Mineral mass (kg)	12	2.4	0.5	117	2.5	0.6
Protein mass (kg)	12	6.8	1.1	117	6.9	1.5
Protein:mineral ratio	12	2.9	0.4	117	2.9	0.4

Abbreviations: 4C, 4-component model; *D*_FFM_, density of FFM; ED, eating disorder; FM, fat mass; FFM, fat-free mass; FMI, fat mass index; FFMI, fat-free mass index; *H*_FFM_, hydration of FFM; SDS, s.d score.

a Significant difference between groups at *P* <0.001.

b Significant difference between group at *P*<0.05.

**Table 3:** Differences in whole-body and regional composition between 12 ED patients versus 117 controls, by general linear model

	*Adjusted age*			*Adjusted age, height, puberty*		
	*Mean*	*s.e.*	P-*values*	*Mean*	*s.e.*	P*-values*
*Whole-body composition*						
FM (kg)	−8.3	2.0	<0.001	−7.7	2.0	<0.001
FFM (kg)	−5.9	1.6	<0.001	−4.0	1.1	<0.001
Mineral mass (kg)	−0.5	0.1	<0.001	−0.3	0.1	0.001
Protein mass (kg)	−0.9	0.3	0.005	−0.6	0.3	0.028
*H*_FFM_ (%)	−0.5	0.5	0.3	−0.4	0.5	0.4
*D*_FFM_ (kg/l)	0.000	0.002	0.8	0.001	0.001	0.6
Protein:mineral ratio	0.2	0.1	0.2	0.1	0.1	0.4
						
*Regional composition*						
Trunk fat (kg)	−4.4	1.2	<0.001	−4.0	1.0	<0.001
Trunk non-bone lean (kg)	−1.6	0.7	0.015	−0.8	0.5	0.089
Trunk bone (g)	−174	40	<0.001	−124	30	<0.001
Arm fat (kg)	−1.0	0.2	<0.001	−0.9	0.2	<0.001
Arm non-bone lean (kg)	−0.3	0.2	0.11	−0.5	0.2	0.002
Arm bone (g)	−7	19	0.7	−31	10	0.003
Leg fat (kg)	−3.0	0.8	<0.001	−3.5	0.8	<0.001
Leg non-bone lean (kg)	−0.6	0.6	0.32	−1.1	0.4	0.003
Leg bone (g)	−40	53	0.4	−85	28	0.003

Abbreviations: *D*_FFM_, density of FFM; ED, eating disorder; FMI, fat mass index; FFMI, fat-free mass index; *H*_FFM_, hydration of FFM.

Differences calculated as: ED−control.

**Table 4:** Evaluation of the accuracy of DXA for body composition assessment in ED patients and controls, using the 4C model as the reference method

	*Bias*					*Correlation*	
	*Mean*		*Limits of agreement*		P*-values*	r	P*-values*
	*(kg)*	*(%)*	*(kg)*	*(%)*			
*ED patients*							
FM	0.4	4.6	±3.1	±36.4	0.4	−0.40	0.2
FFM	−0.3	−0.9	±3.2	±9.4	0.5	0.19	0.5
FMI	0.2	4.6	±1.2	±36.4	0.4	−0.36	0.2
FFMI	−0.1	−0.9	±1.2	±9.4	0.5	0.25	0.4
*Controls*							
FM	1.1	7.4	±2.6	±23.2	<0.001	0.37	<0.001
FFM	−1.2	−3.2	±2.5	±6.2	<0.001	−0.48	<0.001
FMI	0.4	7.4	±1.0	±23.2	<0.001	0.23	0.011
FFMI	−0.5	−3.2	±0.9	±6.2	<0.001	−0.41	<0.001

Abbreviations: 4C, 4-component model; DXA, dual X-ray absorptiometry; FM, fat mass; FMI, FM index; FFM, fat-free mass; FFMI, FFM index.

Bias calculated as DXA value−4C value. Limits of agreement calculated as twice the s.d. of the bias. Correlation between magnitude of bias and the mean value by both techniques.
