# Supplementary material for: Body composition in young female eating-disorder patients with severe weight loss and controls: evidence from the four-component model and evaluation of DXA
Source: Eur J Clin Nutr. 2015 Jul 15;69(12):1330–5. doi: 10.1038/ejcn.2015.111 (PMC4672328; doi:10.1038/ejcn.2015.111)
Supplement: Supplementary Information [file ejcn2015111x1.doc]

**Supplementary Online Table 1: Description of regional body composition standard deviation scores by DXA**

**Eating disorders (n = 13) Controls (n = 117) Significance**

Mean SD Mean SD p

Arm fat SDS -1.41 0.84 0.016 0.99 <0.0001

Arm lean SDS -1.57 0.79 -0.38 1.08 <0.0001

Leg fat SDS -1.60 0.77 0.06 0.96 <0.0001

Leg lean SDS -1.35 0.93 -0.42 0.94 0.001

Trunk fat SDS -1.14 0.69 0.14 1.00 <0.0001

Trunk lean SDS -0.97 0.93 -0.25 1.02 0.016

Groups compared using independent samples t-test
